# Effect of *Lachancea thermotolerans* on the Formation of Polymeric Pigments during Sequential Fermentation with *Schizosaccharosmyces pombe* and *Saccharomyces cerevisiae*

**DOI:** 10.3390/molecules23092353

**Published:** 2018-09-14

**Authors:** Carlos Escott, Antonio Morata, Jorge M. Ricardo-da-Silva, María Jesús Callejo, María del Carmen González, José Antonio Suarez-Lepe

**Affiliations:** 1 enotecUPM. Chemistry and Food Technology Department, School of Agronomic, Food and Biosystems Engineering. Universidad Politécnica de Madrid, Av. Puerta de Hierro 2, 28040 Madrid, Spain; antonio.morata@upm.es (A.M.); mj.callejo@upm.es (M.J.C.); carmen.gchamorro@upm.es (M.d.C.G.); joseantonio.suarez.lepe@upm.es (J.A.S.-L.); 2 LEAF-Linking Landscape, Environment, Agriculture and Food, Instituto Superior de Agronomia, Universidade de Lisboa, Tapada da Ajuda, 1349-017 Lisboa, Portugal; jricardosil@isa.ulisboa.pt

**Keywords:** malvidin, polymeric pigments, non-*Saccharomyces*, red wine, flavanols

## Abstract

Anthocyanins in red grape musts may evolve during the winemaking process and wine aging for several different reasons; colour stability and evolution is a complex process that may depend on grape variety, winemaking technology, fermentative yeast selection, co-pigmentation phenomena and polymerization. The condensation of flavanols with anthocyanins may occur either with the flavylium ion or with the hemiacetal formation in order to produce oligomers and polymers. The kinetics of the reaction are enhanced by the presence of metabolic acetaldehyde, promoting the formation of pyranoanthocyanin-type dimers or flavanol-ethyl-anthocyanin structures. The experimental design carried out using white must corrected with the addition of malvidin-3-*O*-glucoside and flavanols, suggests that non-*Saccharomyces* yeasts are able to provide increased levels of colour intensity and larger polymeric pigment ratios and polymerization indexes. The selection of non-*Saccharomyces* genera, in particular *Lachancea thermotolerans* and *Schizosaccharomyces pombe* in sequential fermentation, have provided experimental wines with increased fruity esters, as well as producing wines with potential pigment compositions, even though there is an important reduction of total anthocyanins.

## 1. Introduction

There are different factors that determine the colour evolution and colour stability of red wines, including grape variety as a sole source of anthocyanins [1]; practices during the winemaking process such as tannin addition [2] or cold maceration [3]; the selection of fermentative yeast strains that promote the formation of stable pigments through the production of metabolites, such as pyruvic acid or acetaldehyde [4], or that promote changes in pigment composition through the adsorption of anthocyanins through the yeast cell walls [5]; and co-pigmentation phenomena [6].

Yeast metabolism may lead to different values of metabolic precursors during must fermentation for the formation of pyranoanthocyanins, oligomeric and polymeric pigments. Non-*Saccharomyces* yeasts have been shown to produce a concentration of pyruvic acid and acetaldehyde that is different from *Saccharomyces cerevisiae* [7,8,9,10]. Escott et al. [11] found a higher concentration of stable pigments produced during fermentation with non-*Saccharomyces* yeasts in comparison to pure fermentations with *S. cerevisiae*.

Although the reactivity of the flavylium ion may be different from that of the hemiacetal form, both molecules interact with flavanols to form oligomers in suspension; in fact, according to Es-Safi and Cheynier [12], dimers are also formed from the condensation of a hemiacetal moiety with flavanols. The latter products, initially belonging to the non-coloured fraction, would be displaced into the coloured form through a co-pigmentation equilibrium [13] that takes place in typical red wine pH and acid aqueous solutions. The dimers formed by the nucleophilic addition of anthocyanins in their hemiacetal form to produce flavanol-anthocyanin adducts (F-A^+^) were reported by Salas et al. [14]. These non-acetaldehyde-mediated oligomers are produced when the flavanol carbocation (F^+^) interacts with positions C6 and/or C8 of anthocyanins

Oligomer pigments are more stable, not as a consequence of their larger molecular weight, but due to the fact that position 4, C4 in the pyran ring of anthocyanins, is protected by the formation of ethyl-linked oligomeric derivatives or the formation of pyranoanthocyanins [15]. Dimeric pigments formed through the condensation of two malvidin-3-*O*-glucoside molecules were found in wine-like solutions and red wines and described by Atanasova et al. [16]. The dimer helped prove the existence of acetaldehyde-mediated, self-condensed anthocyanins. The structure described was formed by two malvidin-3-*O*-glucoside moieties linked by an acetaldehyde molecule at positions C8 in both anthocyanins. The colour retention observed in pyranoanthocyanins at higher pH values could be explained by the fact that these pigments may not undergo hydration reactions [17], which explains their higher stability against monomeric anthocyanins.

The aim of the work was to use white grape must to monitor the condensation of malvidin-3-*O*-glucoside with four different flavanols during fermentation with pure *S. cerevisiae* culture as control, and sequential fermentations with non-*Saccharomyces* yeasts. The flavanols tested in this experiment design were (+)-catechin, procyanidin B2, procyanidin C1 and procyanidin A2.

## 2. Results and Discussion

### 2.1. Yeast Growth and Metabolite Production

**Figure 1:**
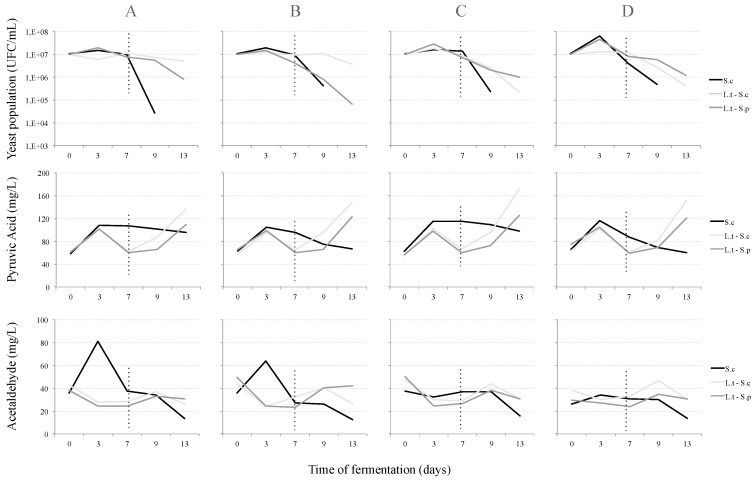
Yeast population growth. Data from acetaldehyde and pyruvic acid concentrations during fermentation. Analyses performed with gas chromatography (GC-FID) (acetaldehyde), enzymatic analyser (pyruvic acid) and plate counting (cells growth). Each point represents mean values (*n* = 3). Dotted lines indicate the sequential yeast inoculation. (**A**) CAT, (**B**) PB2, (**C**) PC1 and (**D**) PA2.

The growth kinetics of the yeast populations can be seen in Figure 1. There were three different fermentation trials, one of which was the pure culture of Sc and two other sequential fermentations with Lt + Sc and Lt + Sp. It was found that within three days, Sc as well as Lt achieved maximum growth with 6.4 × 107 CFU/mL and 4.5 × 107 CFU/mL, respectively, but neither case had a stationary phase, as had been observed previously for pure culture and co-fermentations of *Saccharomyces cerevisiae* by Sadineni et al. [9]; a rapid decrease in Sc populations after day 7 was observed, resulting in an average of 1 × 105 CFU/mL at day 9. With regard to the sequential fermentation with Sc, there was a slight increase and steady growth observed in the CAT and PB2 treatments, while for the other treatments, including Sp, there was a slower decrease in yeast populations over time, until the population numbers ranged between 1 × 105 and 1 × 106 CFU/mL after day 13; this might be due to the fact that the increasing concentration of ethanol makes it difficult for *Lachancea* spp., and several other non-*Saccharomyces* yeasts such as *M. pulcherrima*, *T. delbrueckii* and *C. zemplinina* [18], to survive over fermentation time.

The production of fermentative metabolites followed the kinetic growth, reaching a maximum concentration after 72 h, although no increases were observed in the production of these metabolites after sequential inoculation. Pyruvic acid reached the maximum concentration in all treatments for both yeasts in the initial phase: Sc reached 116 mg/L and Lt reached 105 mg/L. With regard to acetaldehyde production, the maximum concentration was observed in CAT and PB2 treatments for pure culture Sc (81.1 mg/L and 63.6 mg/L respectively), and steady amounts for PC1 and PA2. The concentration of pyruvic acid increased after the sequential inoculation of yeasts Sc (>140 mg/L) and Sp (>110mg/L) in all treatments, with the exception of pure culture Sc, where a steady phase and slight decrease was observed after 72 h and across the remaining fermentation time. On the other hand, when using Lt, the concentration of acetaldehyde decreased during the initial fermentative phase. This is likely due to the fact that the production of acetaldehyde is proportional to the sugars fermented [5], and also because Lt decreases its metabolism when the ethanol concentration increases. After the sequential inoculation of yeasts (Sc and Sp), acetaldehyde production increased and reached its peak at day 9 (Figure 1) with values of ca. 40 mg/L.

### 2.2. Ethanol, Acidity and Sugar Content

Sequential fermentation carried out with the species Lt-Sp resulted in residual sugar (both glucose and fructose combined) higher than 4 g/L, which led to a lower ethanol content compared to the other fermentations (Lt-Sc and pure Sc), however, statistical differences were not evident (Table 1). The differences found in the acidic composition depended on the yeast species used to carry out the fermentations and was independent of the flavanols used in each treatment. Volatile acidity, as well as total acidity were statistically higher for the fermentations carried out with Sc (>0.6 g/L), while the Lt-Sp combination had the lowest concentrations for both parameters; these values were similar to those observed by Sun, Gong, Jiang, and Zhao [19], with more than 0.55 g/L for pure culture fermentation with the strain of *S. cerevisiae* and mixed fermentation with a strain of *M. pulcherrima*. On the other hand, lactic acid was statistically higher for those fermentations where *Lachancea thermotolerans* was used as the starter culture (Lt-Sc and Lt-Sp) compared to pure Sc fermentation. The malic acid content was statistically lower for the Lt-Sp pair and therefore the pH values were higher in these fermentations compared to fermentations with Sc. The demalication observed during the last few trials was performed by *Schizosaccharomyces pombe*, through which malic acid was transformed into ethanol and CO_2_ [10].

### 2.3. Fermentative Volatiles

A summary of the fermentative volatiles is given in Table 2, and have been grouped into four different categories: higher alcohols, carbonyl compounds, fruity esters and total volatiles. Higher alcohols, except 2,3-butanediol, had in all trials concentrations below the 350 mg/L threshold [20,21], therefore it was not expected that they would contribute to an aromatic defect of the wines. The fermentative Lt-Sc yeast combination produced the highest concentration of total higher alcohols (between 227.7 ± 5.3 and 253.9 ± 8.3 mg/L), while fermentations done with Sc produced the lowest concentrations (between 135.9 ± 4.4 and 179.8 ± 4.6 mg/L). Acetoin, diacetyl and ethyl acetate were grouped as carbonyl compounds; when these molecules are higher in concentration than their threshold value, it can result in off-aromas in red wine. Ethyl acetate is a compound with a fruity aroma and can also be linked to solvent or glue in high concentrations [22]; according to Benito, Morata, Palomero, Gonzalez, and Suárez-Lepe [23], acetoin, with a threshold of 10 mg/L, and diacetyl, with a threshold in wines of 2 mg/L, could be responsible for buttery aromas. Regarding single molecules, in contrast to the values of ethyl acetate higher than 125 mg/L obtained in sequential fermentations when using *Metschnikowia pulcherrima* [11], the concentrations obtained in these trials were between 24.4 ± 0.1 mg/L and 49.4 ± 1.6 mg/L; wines made with the Lt-Sp combination were lower, while Lt-Sc wines were the highest. These concentrations are similar to the values observed by Loira et al. [7] for fermentations involving *Torulaspora delbrueckii* and *Saccharomyces cerevisiae,* with values between 6 mg/L and 30 mg/L. In terms of diacetyl, the fermentative pair Lt-Sc had higher values that were statistically different; the values ranged from 5.2 ± 0.3 mg/L to 11.3 ± 0.1 mg/L, all of which were higher than the threshold and may be noticed by the consumer. According to Bartowski and Henschke [24], diacetyl can provide a buttery aroma and flavour to a series of fermented foods and beverages, but is also an intermediate in the production of acetoin and the further production of 2,3-butanediol. The concentration of acetoin produced was slightly higher than the other fermentations, yet statistically significant for the fermentative Lt-Sp yeast combination with values between 7.5 ± 0.2 mg/L and 8.1 ± 0.3 mg/L, meaning all wines were lower in acetoin than the threshold. The values for these three compounds together were statistically higher for the combination Lt-Sc, especially due to the higher concentrations of ethyl acetate and diacetyl produced during fermentation.

Fruity esters were present in higher concentration in wines produced with Lt-Sp combinations, followed by Lt-Sc wines, compared to Sc wines. As with higher alcohols and carbonyl compounds, the fruity ester concentration seems to depend on the fermentative yeast used for wine fermentation and not on the treatment. The highest concentrations were obtained with two non-*Saccharomyces* yeasts yielding concentrations between 23.6 ± 0.7 mg/L and 27.2 ± 1.7 mg/L. Among the fruity esters evaluated, coffee or strawberry and raspberry aromas are produced by ethyl lactate [25]; rose, honey and apple aromas may be derived from 2-phenylethyl acetate [26], and according to Boss et al. [27], the overall contribution of esters to wine is expected to be fruity and flowery.

The concentration of total volatiles mostly consisted of 2,3-butanediol. The total fermentative volatiles were higher in wines produced with Sc given that this yeast produced higher concentrations of 2,3-butanediol (>1000 mg/L), compared to the concentration of higher alcohols and carbonyl compounds, which was higher in wines fermented with Lt-Sc. Wines fermented with Lt-Sp were higher in fruity esters. In these trials, Sc yeasts reduced acetoin to 2,3-butanediol to a larger extent than for the other fermentations, as was noted in fermentations performed with genetically engineered *Saccharomyces cerevisiae* strains [28]. This was due to the enzyme acetoin reductase, which reduces acetoin to 2,3-butanediol, involved in the metabolic pathway from pyruvate [29].

### 2.4. Characterization of Pigments

The cell walls of the yeast strains may adsorb anthocyanins to different extents [30], with some of these adsorbing up to three times more than others [31]. This suggests that the reduction of pigments during fermentation may be conditioned to the fermentative strains selected. This is the case observed in this experiment, especially when using the fermentative strains Lt-Sp, where a significant reduction in the concentration of malvidin-3-*O*-glucoside and total pigments was observed (Table 3). The use of Lt in the initial phase may lead to the first reduction in total pigments, later accentuated during a sequential fermentation with Sp. The CAT treatment was the only one where monomeric malvidin derivatives, other than malvidin-3-*O*-glucoside, were found; the pigment identified in these trials was malvidin-3-*O*-(*p*-coumaroyl)-glucoside with molecular ion [M]^+^ (*m*/*z*) 639 and fragment ion(*m*/*z*)331 [32].

Pyranoanthocyanins are shown in Table 3 in the vitisins and vinylphenolics groups. Regarding vitisin content, the pigment malvidin-3-*O*-glucoside acetaldehyde (Vitisin B) with molecular ion [M]^+^ (*m*/*z*) 517 and fragment ion with (*m*/*z*) 355 [33] was identified in all trials with no significant difference among them; nonetheless, the concentration of vitisin seems lower for sequential fermentation trials in treatment PA2 (Table 3). According to He et al. [34], the malvidin-3-*O*-glucoside-4-vinylphenol with molecular ion [M]^+^ (*m*/*z*) 609 was the only vinylphenolic pigment identified in the trials where Sc was used as pure culture and sequential fermentation. The concentration in the pure culture trials ranged between 0.25 and 0.35 mg/L, while the concentration in the trials with the fermentative strains Lt-Sc was between 0.18 and 0.22 mg/L (Table 3). The fermentative pair Lt-Sp did not produce vinylphenolics due to a lack in hydroxycinnamate decarboxylase activity; this enzymatic activity is associated with the selected Sc strain and is the result of the condensation of malvidin-3-*O*-glucoside and the decarboxylation of p-coumaric acid [5]. Although Suárez-Lepe and Morata [35] have reported hydroxycinnamate decarboxylase activity (HCDC) in non-*Saccharomyces* spp., there were no vinylphenolic pigments present in the wines produced with Lt-Sp yeasts in any of the treatments evaluated.

Polymeric pigments were formed during fermentation in all treatments and with all fermentative strains (Table 4). In treatment CAT, the isomers malvidin-3-*O*-glucoside-vinyl-catechin and malvidin-3-*O*-glucoside-vinyl-epicatechin with molecular ion [M]^+^ (*m*/*z*) 805 [36] were found in similar concentrations in trails fermented with Sc, while in sequential fermentations with Lt-Sp, the quantity of the epicatechin derivative was higher. The third oligomer found in this treatment was malvidin-3-*O*-(*p*-coumaroyl)-glucoside-8-ethyl-(epi) catechin with molecular ion [M]^+^ (*m*/*z*) 955 and fragment ions with (*m*/*z*) 803 and 647 [34]; this structure corresponds to an ethyl-linked flavanol–anthocyanin dimer. PB2 treatment produced slightly higher concentrations of polymeric pigments with statistical significance for the fermentative strains Lt-Sp; the polymeric pigments identified in this treatment were malvidin-3-*O*-glucoside-epicatechin with molecular ion [M]^+^ (*m*/*z*) 781 and fragment ion (*m*/*z*) 619, malvidin-3-*O*-glucoside-4-ethyl-epicatechin with molecular ion [M]^+^ (m/z) 809, malvidin-3-*O*-glucoside-4-vinyl-epicatechin with molecular ion [M]^+^ (*m*/*z*) 805 and malvidin-3-*O*-glucoside-4-vinyl-diepicatechin with molecular ion [M]^+^ (*m*/*z*) 931 [34]. The latter three pigments correspond to a flavanyl–pyranoanthocyanin structure, and the last pigment may correspond to the condensation of a malvidin unit with procyanidin B2, a trimer that is abundant in trials using the Sc strain in pure culture fermentation. The maximum absorption wavelength of these polymeric pigments was between 530 nm and 540 nm.

Under the PC1treatment, three dimers and a trimer were identified: the dimers were the isomers malvidin-3-*O*-glucoside-8-ethyl-catechin and malvidin-3-*O*-glucoside-8-ethyl-epicatechin with molecular ion [M]^+^ (*m*/*z*) 805 [34,36], and molecular structure flavanol-anthocyanin ethyl linked and the non-acetaldehyde mediated malvidin-3-*O*-glucoside-(epi) catechin with molecular ion [M]^+^ (*m*/*z*) 781 and fragment ion (*m*/*z*) 619 [34]; the trimer found, with a slightly higher concentration in trials with strains Lt-Sp, was the malvidin-3-*O*-glucoside-4-vinyl-diepicatechin with molecular ion [M]^+^ (*m*/*z*) 931 [32]. There were no pigments identified from the direct condensation of procyanidin C1 (trimer) with malvidin-3-*O*-glucoside. The fourth treatment, PA2, produced the least concentration of oligomeric pigments, especially the fermentative strain Sc in pure culture fermentation with 0.09 ± 0.01 mg/L. The polymeric pigments found in this treatment were malvidin-3-*O*-glucoside-epicatechin with molecular ion [M]^+^ (*m*/*z*) 781 and fragment ion (*m*/*z*) 619, the flavanol pyranoanthocyanin dimer malvidin-3-*O*-glucoside-4-vinyl-epicatechin with molecular ion [M]^+^ (*m*/*z*) 805 and flavanol pyranoanthocyanin trimer malvidin-3-*O*-glucoside-4-vinyl-diepicatechin with molecular ion [M]^+^ (*m*/*z*) 931 [32].

The direct condensation of the flavanol–anthocyanin dimers occurred at slower and lower yielding rates [37] than for the polymeric pigments formed when acetaldehyde was present. In this way, Dallas, Ricardo-da-Silva, and Laureano [38] have also documented the direct condensation of procyanidin B2 with malvidin-3-*O*-glucoside in the absence of acetaldehyde. Most of the polymeric pigments identified in this experimental design were mediated by acetaldehyde. The oligomer formation was not restrictive to having one moiety of anthocyanin and another of flavanol; two flavanol units may also condense in the presence of acetaldehyde and the anthocyanin may even be in hemiacetal form; from these polymeric pigments formed, the ethyl-flavanol oligomers are less stable [12]. The co-pigmentation equilibrium phenomena may increase the colour of wine, displacing some of the colourless anthocyanins into coloured oligomers [13]. Three peaks were labelled as unidentified, as the molecular weight does not correlate to the expected molecular structures. Unidentified peaks 1 and 3 occurred in treatments CAT and PB2, while all three were present in treatments PC1 and PA2.

**Figure 2:**
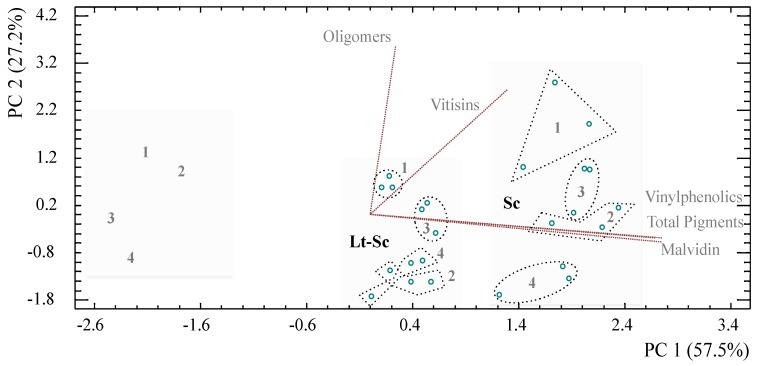
Principal Component Analysis (PCA) for pigments determination in experimental trials. The clusters identified correspond to the fermentative yeasts used (block font). The numbers refer to each of the four treatments assessed: (1) CAT; (2) PB2; (3) PC1, and; (4) PA2.

The contribution of the yeast strains and the different treatments to the evolution and stability of the pigments can be seen in a PCA chart in Figure 2. Two principal components explain the 84.7% variability of the experimental design. Component 1 had positive contributions from malvidin-3-*O*-gucoside, total pigments and vinyphenolics while Component 2 had positive contributions from polymeric pigments and vitisins. In this way, there are three different clusters observed with respect to Component 1, where those trials with more pigments are located to the right side of the chart and correspond to pure culture fermentation with Sc, while trials with the least concentration are located to the left; this behaviour was observed by Escott et al. [11] for other non-*Saccharomyces* spp. including *T. delbrueckii* and *M. pulcherrima*. Regarding Component 2, the contribution of the treatments to the pigment formation can be seen for each fermentative media; the CAT treatment produced the highest concentration of oligomeric pigments in all three clusters, as previously observed in the results of the HPLC-DAD-ESI/MS analysis, followed by PB2 treatment for trials with Lt-Sp (Figure 2–left side) and by the PC1 treatment for trials Sc and Lt-Sc.

### 2.5. Colour and Polyphenol Assessment 

Spectrophotometry, in particular UV-Vis, was used to determine the colour parameters as well as polyphenols due to the ability of anthocyanins and flavanols to absorb at different wavelengths in function of their molecular structure. Table 5 summarizes all parameters obtained from the absorption at wavelengths in both the UV and the visible spectra. The colour intensity and hue showed greater values for the trials fermented with the pair Lt-Sp for all treatments; there was also a slight increase in the values of these parameters in the following order of fermentative yeasts Sc < Lt-Sc < Lt-Sp. The anthocyanin ionization degree (Table 5) was also higher for the fermentative pair Lt-Sp, meaning that the fraction of pigments with flavylium form was higher, and therefore the observed colour may be more intense. The concentration of pigments measured with this technique was inverse to the colour intensity, meaning that the wines fermented with Sc had a larger amount of pigments at the end of fermentation, and, as found with high performance liquid chromatography coupled with mass spectrometer (HPLC-DAD-ESI/MS), the concentration of malvidin-3-*O*-glucoside was higher in these wines followed by the wines where Lt-Sc was used. The trials with the lowest concentration of polymeric pigments and total pigments corresponded to those fermented with Sp. The trend of these values differed from that observed with HPLC-DAD-ESI/MS, making evident the larger measuring range of the UV-Vis technique.

With regard to chemical age (ChA) and polymerization index (PI) (Table 5), the values were higher for the fermentative pair Lt-Sp as observed for hue (N), mainly due to the fact that the ratio of oligomeric pigments to total pigments was higher for these trials even though the concentration of total pigments was shorter, as found by Chen et al. [39]. According to these authors, the concentration of monomeric and total pigments decreased when using Sp as the fermentative yeast, but the fraction of oligomeric pigments increased. The fact of having more oligomeric pigments, and therefore higher concentrations of ChA, suggests an increase in compounds with absorption at shorter wavelengths, similar to those pigments found in aged wines. The values of the total polyphenols index (TPI) were statistically different for the trials Lt-Sp, with a slightly higher numerical difference from the other trials.

A Pearson correlation plot for the pigments and colour parameters is shown in Table 6. The plot confirms the results previously discussed. For the pigments and colour parameters, the correlation indicates a strong and very strong negative relationship between malvidin and vinylphenolics with most indexes (I, N, TPI, ChA, PI and PP), while the relationship was very strongly positive between these pigments and the total pigments index (TP). For vitisin and oligomers, the correlation with all indexes, except for polymeric pigments, was not significant at 5%; the correlation between these pigments and the polymeric pigment index (PP) was strongly positive. It can be observed that the higher the amount of malvidin and vinylphenolics observed at the end of the fermentation, the lower the index obtained. 

## 3. Materials and Methods

### 3.1. Yeast Strains and Yeast Population Growth

The species *Lachancea thermotolerans* (Lt) strain CHKt 421 (Viniflora^®^, Chr-Hansen) was used in sequential fermentation with the *Saccharomyces cerevisiae* (Sc) strain 7VA (EnotecUPM, Madrid, Spain) and the *Schizosaccharomyces pombe* (Sp) strain 938 (IFI, SCIC, Madrid, Spain). A pure-culture fermentation with the yeast *S. cerevisiae* was used as a control trial. All yeasts were firstly grown in stripe, and later in liquid YPD media (yeast eXtract, peptone and deXtrose) before fermentation for 48 h at 25 °C to reach a population in the pre-inoculum of 1.00 × 10^7^ and 1.06 × 10^7^ CFU/mL by count plates for the species Lt and Sc, respectively. The sequential fermentations were performed using the Sc and Sp species, grown in the same liquid YPD media at 25 °C for 48 h to reach a population of 6.01 × 10^6^ and 9.80 × 10^5^ CFU/mL in the respective pre-inoculums. Obtaining 500 μL from the fermenter flasks at days 3, 7, 9 and 13 followed the yeast growing kinetics; the sequential fermentation was done at day 7. The yeast populations were measured by plate counting. The liquid YPD growing media was prepared with 1% yeast eXtract (Laboratorios Conda; Madrid, Spain), 2% bacteriological peptone (Laboratorios Conda; Madrid, Spain), and 2% d (+)-glucose anhydrous (Panreac Química; Barcelona, Spain); the liquid YPD media was sterilized in an autoclave at 120 °C for 15 min. The colonies were determined by plate counting using YPD agar and second medium with lysine agar where *S. cerevisiae* was unable to grow. The growth media for plate counting was performed according to Loira et al. [7] for YPD agar at 25 °C for 2 days and at 28 °C for 2 days for lysine phosphate medium plates for non-*Saccharomyces* spp.

### 3.2. Corrected Must for Winemaking

White concentrated must, *Vitis vinifera* L. cv. Airén from the 2016 vintage, was used to prepare the corrected must base. The must, following correction, had a density of 1100 and a pH of 3.4 after adding 0.79 g/L of l (+)-tartaric acid (Panreac Química; Barcelona, Spain). The alcohol potential for this must was eXpected to be 13.8% *v*/*v* (23.4° BriX). The must was sterilized in an autoclave at 100 °C for 1 min in 3 L containers and included a previous addition of malvidin-3-*O*-glucoside and flavanols. Microbiological control analysis showed no presence of either yeast colonies (10° in 100 mL) or mesophilic bacteria in the must prior to the inoculation of the yeasts (t_0_).

Malvidin-3-*O*-glucoside (CAS: 643-84-5) was added to the sterile must to obtain an initial concentration of 40 mg/L; it was divided into 4 250 mL flasks in order to prepare the four different must media. The four must media treatments were named after the flavanols used: (+)-catechin (CAT), procyanidin B2 (PB2), procyanidin C1 (PC1) and procyanidin A2 (PA2). The flavanols (+)-catechin (CAS: 154-23-4 and purity of 99%), procyanidin B2 (CAS: 29106-49-8 and purity of 90%), procyanidin C1 (CAS: 37064-30-5 and purity of >95%) and procyanidin A2 (CAS:41743-41-3) (Cymit Química S.L., Barcelona. Spain) were used at a concentration of 20 mg/L.

Each of the four model solutions were transferred into 60 mL microfermenters after which 750 μL of inoculum of either Sc (trial 1) or Lt (trials 2 and 3) yeast strains were added in triplicate. Microfermenters were sealed with Müller valves and placed at a constant 25 °C after being weighed. After day 7, a second inoculum of 750 μL was added to the Sc yeasts (trials 1 and 2) or Sp (trial 3) to finish fermenting residual sugars and to achieve maXimum fermentative power. Fermentation kinetics were followed until a stable weight was reached. Fermentations spanned 14 days for pure-culture fermentation and up to 21 days for sequential fermentation. 

**Figure 3:**
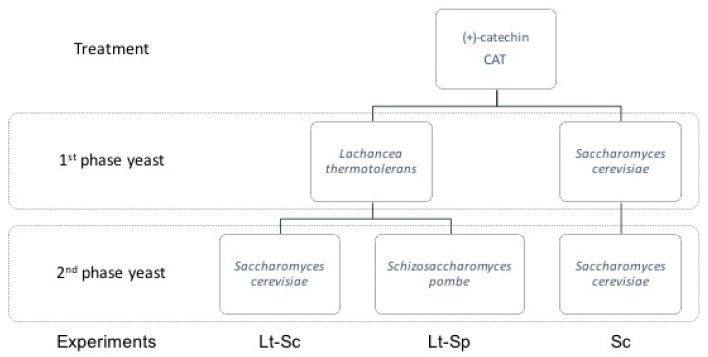
EXperimental design for CAT treatment. Three different trials named Lt-Sc, Lt-Sp and Sc resulted from the combination of 1st and 2nd phase yeast. The other treatments assessed (PB2, PC1, and PA2) followed the same design.

In this way, the eXperiment was comprised of the following miX design: four model solution treatments fermented with Sc as a control (trial 1); a second trial that comprised the use of Lt in sequential fermentation with Sc, and; a third trial had Lt in sequential fermentation with Sp. This resulted in 12 different fermentation trials by triplicate. As an eXample, Figure 3 shows the first three fermentation trials corresponding to the CAT treatment.

### 3.3. Main Wine Parameters

The main parameters such as residual sugars, malic acid, lactic acid, total acidity, volatile acidity and pH values were measured by Fourier transform infrared spectroscopy (FTIR) with the OenoFoss™ equipment (FOSS Iberia, Barcelona, Spain) to characterize wines after fermentation. Pyruvic acid production during fermentation was followed up with an enzymatic analysis by spectrophotometric determination with the use of the Analyzer Y15 (Biosystems, Barcelona, Spain) at times 0, 3, 7, 9 and 13 days and in triplicate. The enzymatic reagent used for the analysis was the 12826-Pyruvic acid (Biosystems, Barcelona, Spain), containing d-Lactate dehydrogenase.

### 3.4. Fermentative Volatile Compounds

A gas chromatography with flame ionization detector (GC-FID) was used for the determination of volatile compounds. The chromatograph used was an Agilent Technologies™ 6850 (Palo Alto, CA, USA) with a DB-624 column (60 m × 250 μm × 1.4 μm). The injector temperature was 250 °C and the detector temperature was set to 300 °C. The temperature went from a steady 40 °C for 5 min to °C at intervals of 10 °C /min. This temperature was maintained for 5 min. Hydrogen was used as a carrier gas at a 2.2 L/min flow with a split ratio of 1:10. The identification and quantification of volatile compounds were performed with 100 μL of 4-methyl-2-pentanol (500 mg/L) used as an internal standard [40]. The method comprised the calibration of the column with the following compounds as eXternal standards: acetaldehyde, methanol, 1-propanol, 1-butanol, 2-butanol, isobutanol, 2-methyl-1-butanol, 3-methyl-1-butanol, 2-phenylethyl acetate, 2-phenylethyl alcohol, diacetyl, ethyl acetate, isoamyl acetate, isobutyl acetate, ethyl butyrate, ethyl lactate and heXanol. For acetaldehyde determination, the calibration R^2^ was 0.99973 for concentrations between 5 and 500 mg/L. The detection limit was 0.1 mg/L.

### 3.5. Pigment Characterization

The use of an HPLC with a diode array detector and electro spray ionization coupled with a mass spectroscopy (DAD-ESI/MS) was used to identify and characterize malvidin-3-*O*-glucoside, vitisin B, malvidin-3-*O*-glucoside acyl derivatives and polymeric pigments with malvidin-3-*O*-glucoside; the HPLC was a Agilent Technologies™ 1100 (Palo Alto, CA, USA) chromatograph with a column RP KineteX C18 100Å (100 × 4.6 mm; 2.6 μm) (PhenomeneX, Torrance, CA, USA). The eluents used comprised eluent A (water/formic acid 95:5 *v*/*v*) and eluent B (methanol/formic acid 95:5 *v*/*v*), with the following gradient of eluent B (0.8 mL/min): from 20% to 50% from 0 min to 27 min; 50% from 27 min to 28 min, and finally, from 50% to 20% from 28 min to 29 min until a steady state was reached. According to Loira et al. [7], malvidin-3-*O*-glucoside has been used as an eXternal standard at a wavelength of 525 nm for the quantification of all pigments, while the identification was carried out with mass spectrometry positive scanning from 100 to 1500 *m*/*z* from 0 min to 23 min. The detection limit was set to 0.1 mg/L [41].

### 3.6. Colour Assessment, Total Anthocyanins and Total Phenolics

The colour intensity (I) as well as colour shade (N), anthocyanin ionization degree (α), total phenolics (TPI), chemical age (ChA), polymerization indeX (PI), polymerized pigments (PP) and total pigments (TP) of red wine samples were determined through the use of a UV-visible spectrometer Unicamp™ UV4 (Algés, Lisbon, Portugal) using 1 mm and 1 cm path length cuvettes following the Somers and Evans [42] methodology. Colour intensity (I) is eXpressed as the sum of absorbance at maXimal wavelengths A420 nm, A520 nm and A620 nm; colour hue (N) is obtained from the ratio A420 nm/A520 nm; anthocyanin ionization degree (α) with the formula [A520 nm − A520 nm_SO2_/A520_HCl_ − 5/3 A520 nm_SO2_] × 100%; total phenolics (TPI) is the indeX obtained from multiplying A280 nm × 10 (dilution correction); chemical age (ChA) is eXpressed as the ratio of A520 nm_SO2_/A520 nm_HCl_; polymerization indeX (PI) obtained from ChA × 100; polymerized pigments (PP) as the absorption A520_SO2,_ and; total pigments (TP) as the absorption A520_HCl_.

### 3.7. Statistical Analysis

Means and standard deviations were calculated and differences eXamined using ANOVA and the least significant difference (LSD) test. All calculations were made using the PC Statgraphics v.XI software (Graphics Software Systems, Rockville, MD, USA). Significance was set at *P* < 0.05.

## 4. Conclusions

The use of (+)-catechin in musts produced a higher concentration of oligomeric pigments, most of which are linked to acetaldehyde. The use of both *Saccharomyces cerevisiae* and *Schizosaccharomyces pombe* in sequential fermentation increased the production of acetaldehyde and pyruvic acid toward the end of the fermentation process, making possible the formation of stable pigments during this stage. Therefore, the colour of wines is a result of the concentration of monomeric pigments and the oligomeric fraction formed during fermentation. The different treatments, meaning the addition of flavanols to musts before fermentation, did not seem to have an influence on the metabolic pathways for fermentative volatile production for samples with the same fermentative conditions; most of the differences in fermentative volatiles found in wines were related to the yeasts used for fermentation. Less 2,3-butanediol and more fruity esters were formed when using non-*Saccharomyces* yeasts. Not only the aromatic profile, but also the colour observed in the wines changed as a result of the nature of the fermentative strains used, some of which may have had higher pigment adsorption on their cell wall structure.

## Figures and Tables

**Table 1:** Ethanol, acidity and sugar content in wine samples. Results are shown by treatment. The statistical analysis was performed by parameter (columns) and the different letters indicate significant statistical difference (LSD method with method with 95% confidence level). Means ± standard deviation (STD) (*n* = 3).

Treatment	EtOH % *v*/*v*	pH	Malic Acid g/L	Volatile Acidity g/L	Lactic Acid g/L	Total Acidity g/L	Glucose g/L	Fructose g/L
*CAT*								
Sc	14.2 ± 0.2 a	3.6 ± 0.0 c	2.0 ± 0.1 ab	0.6 ± 0.0 ab	0.5 ± 0.1 b	5.1 ± 0.0 a	0.9 ± 0.2 b	2.0 ± 0.1 b
Lt-Sc	14.0 ± 0.2 a	3.6 ± 0.0 c	1.7 ± 0.1 d	0.2 ± 0.0 c	0.8 ± 0.1 a	4.9 ± 0.1 bc	0.8 ± 0.1 b	1.2 ± 0.2 b
Lt-Sp	13.7 ± 0.1 a	3.8 ± 0.0 a	0.7 ± 0.1 f	0.2 ± 0.0 c	0.8 ± 0.1 a	3.4 ± 0.1 d	3.5 ± 0.2 a	10.8 ± 0.8 a
*PB 2*								
Sc	14.2 ± 0.1 a	3.6 ± 0.0 c	2.0 ± 0.1 abc	0.6 ± 0.0 b	0.6 ± 0.1 b	5.1 ± 0.1 ab	0.9 ± 0.1 b	1.5 ± 0.2 b
Lt-Sc	14.0 ± 0.3 a	3.6 ± 0.0 c	1.7 ± 0.0 d	0.2 ± 0.0 c	0.7 ± 0.0 a	4.8 ± 0.1 c	0.9 ± 0.1 b	1.3 ± 0.1 b
Lt-Sp	13.8 ± 0.3 a	3.8 ± 0.0 a	0.7 ± 0.0 f	0.2 ± 0.0 c	0.7 ± 0.0 a	3.4 ± 0.1 d	3.1 ± 0.5 a	10.2 ± 0.8 a
*PC 1*								
Sc	14.2 ± 0.2 a	3.7 ± 0.0 bc	2.1 ± 0.1 a	0.6 ± 0.1 ab	0.5 ± 0.0 b	5.2 ± 0.1 a	1.0 ± 0.2 b	2.6 ± 1.1 b
Lt-Sc	14.4 ± 0.1 a	3.6 ± 0.0 c	1.8 ± 0.2 bcd	0.2 ± 0.0 c	0.8 ± 0.1 a	4.8 ± 0.1 c	1.0 ± 0.2 b	1.8 ± 0.7 b
Lt-Sp	13.9 ± 0.3 a	3.8 ± 0.0 ab	0.6 ± 0.1 f	0.2 ± 0.0 c	0.8 ± 0.1 a	3.4 ± 0.1 d	2.8 ± 0.8 a	10.1 ± 0.7 a
*PA 2*								
Sc	14.1 ± 0.2 a	3.6 ± 0.0 c	2.0 ± 0.1 abc	0.7 ± 0.0 a	0.5 ± 0.0 b	5.2 ± 0.1 a	0.7 ± 0.2 b	1.8 ± 0.5 b
Lt-Sc	14.1 ± 0.1 a	3.6 ± 0.0 c	1.7 ± 0.1 cd	0.2 ± 0.0 c	0.9 ± 0.0 a	4.9 ± 0.0 c	1.0 ± 0.1 b	1.6 ± 0.3 b
Lt-Sp	13.7 ± 0.2 a	3.8 ± 0.0 a	0.7 ± 0.1 f	0.2 ± 0.0 c	0.9 ± 0.1 a	3.5 ± 0.1 d	3.3 ± 0.4 a	11.0 ± 0.9 a

CAT. (+)-catechin, PB 2: procyanidin B2, PC 1: procyanidin C1, PA 2: procyanidin A2.

**Table 2:** Summary of fermentative volatile compounds quantified by GC-FID. The statistical analysis is given by parameter (columns) and the difference in letters indicates significant statistical difference (LSD method with 95% confidence level). Means ± STD (*n* = 3).

Treatment	Higher Alcohols ^1^ mg/L	Carbonyl Compounds ^2^ mg/L	Fruity Esters ^3^ mg/L	Total Volatiles mg/L
*(+)-Catechin*				
Sc	135.9 ± 4.4 f	42.0 ± 1.7 c	8.7 ± 0.6 c	1316.6 ± 91,4 c
Lt-Sc	253.9 ± 8.3 a	59.8 ± 1.9 a	15.8 ± 0.4 b	1048.6 ± 11.2 d
Lt-Sp	196.1 ± 1.7 c	38.4 ± 0.6 cd	27.1 ± 1.2 a	933.5 ± 7.4 e
*PB2*				
Sc	154.9 ± 4.1 e	46.7 ± 0.8 b	8.4 ± 0,9 c	1615.7 ± 64.0 b
Lt-Sc	227.7 ± 5.3 b	62.2 ± 1.5 a	14.7 ± 0.4 b	1028.1 ± 9.1 de
Lt-Sp	203.0 ± 5.6 c	36.6 ± 1.5 d	23.6 ± 0.7 a	987.7 ± 7.4 de
*PC1*				
Sc	137.3 ± 1.0 f	36.8 ± 0.6 d	7.5 ± 0.6 c	1421.5 ± 53.5 c
Lt-Sc	233.7 ± 3.8 b	58.3 ± 1.0 a	14.0 ± 0.7 b	1047.9 ± 11.7 de
Lt-Sp	209.2 ± 4.8 c	36.5 ± 1.0 d	24.6 ± 0.5 a	981.7 ± 34.4 de
*PA2*				
Sc	179.8 ± 4.6 d	50.5 ± 0.8 b	8.8 ± 0.4 c	1746.0 ± 16.0 a
Lt-Sc	232.0 ± 7.6 b	58.4 ± 1.9 a	17.7 ± 1.6 b	1033.4 ± 18.3 de
Lt-Sp	206.8 ± 2.9 c	38.4 ± 1.8 cd	27.2 ± 1.7 a	1022.0 ± 15.1 de

^1^ Higher alcohols comprise the following compounds: 1-propanol, 2-buntanol, isobutanol, 1-butanol, 2-methyl-1-butanol, 3-methyl-1-butanol,2-phenylethylalcohol; ^2^ Carbonyl compounds comprise: diacetyl, acetoin, ethyl acetate; and ^3^ Fruity esters comprise: isobutyl acetate, ethyl butyrate, ethyl lactate, isoamyl acetate, 2-phenylethyl acetate.

**Table 3:** Summary of pigments found in experimental red wines. The statistical analysis is given by parameter (columns) and the difference in letters indicates significant statistical difference (LSD method with 95% confidence level). Means ± STD (*n* = 3).

Treatment	Malvidin ^1^ mg/L	Vitisins mg/L	Vinylphenolics mg/L	Oligomeric Pigments ^2^ mg/L
*CAT*				
Sc	28.7 ± 1.8 b	0.1 ± 0.0 abc	0.3 ± 0.0 ab	1.0 ± 0.1 a
Lt-Sc	25.9 ± 0.8 c	0.1 ± 0.0 bc	0.2 ± 0.0 e	0.8 ± 0.0 b
Lt-Sp	14.0 ± 0.7 d	0.1 ± 0.0 abc	0.0 ± 0.0 f	0.6 ± 0.1d
*PB 2*				
Sc	29.7 ± 0.3 ab	0.1 ± 0.0 ab	0.4 ± 0.1 a	0.7 ± 0.0 cd
Lt-Sc	26.7 ± 0.6 c	0.0 ± 0.0 c	0.2 ± 0.0 cd	0.6 ± 0.0 d
Lt-Sp	14.7 ± 1.3 d	0.1 ± 0.0 abc	0.0 ± 0.0 f	0.6 ± 0.0 d
*PC 1*				
Sc	30.8 ± 0.5 a	0.1 ± 0.0 a	0.2 ± 0.0 bc	0.7 ± 0.0 c
LtSc	26.4 ± 0.3 c	0.1 ± 0.0 abc	0.2 ± 0.0 de	0.6 ± 0.1 cd
LtSp	13.7 ± 0.6 d	0.0 ± 0.0 bc	0.0 ± 0.0 f	0.4 ± 0.0 e
*PA2*				
Sc	29.9 ± 0.5 ab	0.1 ± 0.0 abc	0.3 ± 0.1 a	0.6 ± 0.1d
LtSc	26.4 ± 0.3 c	0.0 ± 0.0 bc	0.2 ± 0.0 d	0.4 ± 0.0 e
LtSp	14.3 ± 0.6 d	0.0 ± 0.0 bc	0.0 ± 0.0 f	0.4 ± 0.0 e

^1^ Comprises the content of malvidin-3-*O*-glucoside and its derivative malvidin-3-*O*-(6-*p*-coumaroylglucoside). ^2^ Comprises the content of identified polymeric pigments and unidentified peaks described in Table 4. CAT: (+)-catechin, PB2: procyanidin B2, PC1: procyanidin C1, PA2: procyanidin A2.

**Table 4:** Pigments identified with HPLC-DAD-ESI/MS with DAD signal at 525nm.

Compound	t_R_ (min)	[M]^+^ *m*/*z*	Fragment *m*/*z*	λ _max-vis_ (nm)	Reference
Malvidin-3-*O*-glucoside-epicatechin	12.9	781	619	530	[34]
Unidentified Peak 1	13.4	679	-	514	
Malvidin-3-*O*-glucoside-4-vinyl-diepicatechin	13.9	931	-	533	[32]
Malvidin-3-*O*-glucoside-ethyl-epicatechin	14.2	809	-	538	[34]
Unidentified Peak 2	14.3	995	782	538	
Malvidin-3-*O*-(6-*p*-coumaroylglucoside)-8-ethyl-(epi)catechin	14.5	954	852	520	[34]
Malvidin-3-*O*-glucoside-8-ethyl-epicatechin	14.6	809	643	542	[32]
Malvidin-3-*O*-glucoside-4-vinyl-epicatechin	14.9	805	331	540	[36]
Malvidin-3-*O*-glucoside-8-ethyl-catechin	15.3	809	643	538	[34]
Diepicatechin-malvidin-3-*O*-glucoside	16.2	1069	619	506	[34]
Unidentified Peak 3	17.3	829	691	512	

(t_R_) retention time; ([M]^+^) molecular ion; (λ) maximum absorption wavelength.

**Table 5:** Results from UV-Vis determination of colour parameters and polyphenols. The statistical analysis is given by parameter (columns) and the difference in letters indicates significant statistical difference (LSD method with 95% confidence level). Means ± STD (*n* = 3).

Treatment	I AU	N	± %	TPI AU	ChA	PI %	PP AU	TP AU
*CAT*								
Sc	0.78 ± 0.0 bc	1.59 ± 0.0 c	17.76 ± 0.8 bc	13.81 ± 0.1 b	0.13 ± 0.0 bc	12.77 ± 0.6 de	0.13 ± 0.0 bc	1.03 ± 0.1 a
Lt-Sc	0.80 ± 0.0 ab	1.65 ± 0.0 c	30.84 ± 1.8 a	13.41 ± 0.1 b	0.23 ± 0.0 a	23.12 ± 0.6 ab	0.15 ± 0.0 a	0.66 ± 0.0 d
Lt-Sp	0.82 ± 0.0 ab	2.25 ± 0.0 ab	23.53 ± 3.6 b	14.77 ± 0.1 a	0.25 ± 0.0 a	25.27 ± 2.2 a	0.15 ± 0.0 a	0.59 ± 0.0 d
*PB2*								
Sc	0.71 ± 0.1 d	1.59 ± 0.0 c	16.39 ± 0.2 c	13.70 ± 0.5 b	0.12 ± 0.0 c	11.80 ± 0.7 e	0.12 ± 0.0 c	1.02 ± 0.1 ab
Lt-Sc	0.81 ± 0.0 ab	1.64 ± 0.0 c	22.86 ± 2.2 bc	13.39 ± 0.0 b	0.19 ± 0.0 b	19.17 ± 1.1 bc	0.16 ± 0.0 a	0.82 ± 0.1 c
Lt-Sp	0.84 ± 0.0 a	2.17 ± 0.0 b	22.68 ± 4.2 bc	15.04 ± 0.2 a	0.25 ± 0.0 a	24.61 ± 1.3 a	0.16 ± 0.0 a	0.64 + 0.1 d
*PC1*								
Sc	0.74 ± 0.0 cd	1.64 ± 0.0 c	18.49 ± 1.6 bc	13.61 ± 0.1 b	0.13 ± 0.0 bc	12.84 ± 0.6 de	0.12 ± 0.0 c	0.95 ± 0.1 b
Lt-Sc	0.80 ± 0.0 ab	1.65 ± 0.0 c	22.48 ± 1.2 bc	13.36 ± 0.1 b	0.18 ± 0.0 b	18.44 ± 0.4 c	0.15 ± 0.0 a	0.82 ± 0.0 c
Lt-Sp	0.84 ± 0.0 a	2.20 ± 0.1 ab	24.53 ± 2.9 ab	14.64 ± 0.3 a	0.27 ± 0.0 a	26.70 ± 2.7 a	0.16 ± 0.0 a	0.59 ± 0.0 d
*PA2*								
Sc	0.75 ± 0.0 cd	1.64 ± 0.0 c	20.60 ± 1.2 bc	13.81 ± 0.1 b	0.16 ± 0.0 ab	15.84 ± 0.7 cd	0.14 ± 0.0 b	0.85 ± 0.0 c
Lt-Sc	0.82 ± 0.0 ab	1.66 ± 0.0 c	23.22 ± 2.5 b	13.38 ± 0.2 b	0.19 ± 0.0 b	19.05 ± 1.9 c	0.16 ± 0.0 a	0.82 ± 0.1 c
Lt-Sp	0.84 ± 0.0 a	2.26 ± 0.0 a	21.77 ± 2.0 bc	14.86 ± 0.2 a	0.25 ± 0.0 a	25.31 ± 0.9 a	0.16 ± 0.0 a	0.62 ± 0.0 d

I: Colour intensity; N: Hue (N); α: anthocyanin ionization degree; TPI: total polyphenols index; ChA: chemical age; PI: polymerization index; PP: polymerized pigments; TP: total pigments.

**Table 6:** Pearson correlation plot for pigments and colour indexes. X indicates values not significant at 5%. *P*-values below 0.05 indicate statistically significant non-zero correlations. *R* values = +0.7 or higher indicate very strong positive relationship while, *R* values = −0.7 or higher indicate very strong negative relationship.

	Malvidin	Vitisin	Vinyphenolics	Oligomers	I	N	α	TPI	ChA	PI	PP	TP
**Malvidin**		X	0.930	X	−0.799	−0.979	X	−0.890	−0.906	−0.900	−0.660	0.872
**Vitisin**	X		X	0.704	X	X	X	X	X	X	−0.633	X
**Vinyphenolics**	0.930	X		X	−0.848	−0.920	X	−0.783	−0.901	−0.898	−0.696	0.889
**Oligomers**	X	0.704	X		X	X	X	X	X	X	−0.645	X
**I**	−0.799	X	−0.848	X		0.701	0.602	X	0.878	0.882	0.920	−0.816
**N**	−0.979	X	−0.920	X	0.701		X	0.939	0.841	0.834	X	−0.826
**α**	X	X	X	X	0.602	X		X	0.711	0.720	0.667	−0.733
**TPI**	−0.890	X	−0.783	X	X	0.939	X		0.672	0.654	X	−0.649
**ChA**	−0.906	X	−0.901	X	0.878	0.841	0.711	0.672		0.999	0.833	−0.985
**PI**	−0.900	X	−0.898	X	0.882	0.834	0.720	0.654	0.999		0.840	−0.987
**PP**	−0.660	−0.633	−0.696	−0.645	0.920	X	0.667	X	0.833	0.840		−0.788
**TP**	0.872	X	0.889	X	−0.816	−0.826	−0.733	−0.649	−0.985	−0.987	−0.788	

I: Colour intensity; N: Hue (N); α: anthocyanin ionization degree; TPI: total polyphenols indeX; ChA; chemical age; PI: polymerization indeX; PP: polymerized pigments; TP: total pigments.

## Chemical Compounds Studied in This Article

Acetaldehyde (PubChem CID: 177); Malvidin-3-*O*-glucoside (PubChem CID: 443652); Pyruvic acid (PubChem CID: 1060); Vitisin A (PubChem CID: 16131430); Vitisin B (PubChem CID: 16138152); (+)-Catechin (PubChem CID: 73160); Procyanidin B2 (PubChem CID: 122738); Procyanidin C1 (PubChem CID: 169853); Procyanidin A2 (PubChem CID: 124025); Malvidin-3-*O*-glucoside-ethyl-catechin (PubChem CID: 71308233)

## Abbreviations

Lt	Lachancea thermotolerans
Sc	Saccharomyces cerevisiae
Sp	Schizosaccharomyces pombe
CAT	(+)-catechin
PB2	procyanidin B2
PC1	procyanidin C1
PA2	procyanidin A2
I	colour Intensity
N	hue
ChA	chemical age
TPI	total polyphenols indeX
N	hue; ionization degree
PP	polymeric pigments
PI	polymerization indeX
CFU/mL	colony-forming unit per milliliter
